# Personal protective equipment use among dental healthcare personnel during the coronavirus disease 2019 (COVID-19) pandemic and the impact of an educational video in clinical practice

**DOI:** 10.1017/ice.2023.6

**Published:** 2023-09

**Authors:** Lucy C. Vogt, Kimberly A. Reske, Daniel Park, Tracey Habrock Bach, Henry B. Stewart, Olivia G. Arter, Daniel Stoeckel, Heidi M. Steinkamp, Stephen Y. Liang, Michael J. Durkin, Jennie H. Kwon

**Affiliations:** 1 Division of Infectious Diseases, Department of Medicine, Washington University School of Medicine, St. Louis, Missouri; 2 St. Louis University Center for Advanced Dental Education, St. Louis, Missouri; 3 St. Louis Children’s Hospital, St. Louis, Missouri; 4 Department of Emergency Medicine, Washington University School of Medicine, St. Louis, Missouri

## Abstract

**Objective:**

Dental healthcare personnel (DHCP) are at high risk of exposure to coronavirus disease 2019 (COVID-19). We sought to identify how DHCP changed their use of personal protective equipment (PPE) as a result of the COVID-19 pandemic, and to pilot an educational video designed to improve knowledge of proper PPE use.

**Design:**

The study comprised 2 sets of semistructured qualitative interviews.

**Setting:**

The study was conducted in 8 dental clinics in a Midwestern metropolitan area.

**Participants:**

In total, 70 DHCP participated in the first set of interviews; 63 DHCP participated in the second set of interviews.

**Methods:**

In September–November 2020 and March–October 2021, we conducted 2 sets of semistructured interviews: (1) PPE use in the dental community during COVID-19, and (2) feedback on the utility of an educational donning and doffing video.

**Results:**

Overall, 86% of DHCP reported having prior training. DHCP increased the use of PPE during COVID-19, specifically N95 respirators and face shields. DHCP reported real-world challenges to applying infection control methods, often resulting in PPE modification and reuse. DHCP reported double masking and sterilization methods to extend N95 respirator use. Additional challenges to PPE included shortages, comfort or discomfort, and compatibility with specialty dental equipment. DHCP found the educational video helpful and relevant to clinical practice. Fewer than half of DHCP reported exposure to a similar video.

**Conclusions:**

DHCP experienced significant challenges related to PPE access and routine use in dental clinics during the COVID-19 pandemic. An educational video improved awareness and uptake of appropriate PPE use among DHCP.

Coronavirus disease 2019 (COVID-19) is a public health crisis that has affected the entire healthcare community. Although much of the focus of the pandemic has been on acute-care hospitals, the Occupational Safety and Health Administration (OSHA) has identified dental healthcare personnel (DHCP) as being at high risk for work-related COVID-19 exposure due to direct contact with aerosols and respiratory droplets from unmasked patients.^1–3^

The COVID-19 pandemic reinforced the importance of adequate training in personal protective equipment (PPE) to reduce risk of exposure to severe acute respiratory syndrome coronavirus 2 (SARS-CoV-2), the virus that causes COVID-19. Early in the pandemic, many DHCP voiced concerns related to limited knowledge of infection control methods, PPE availability and use, and personal health.^4–6^ Although recommendations and guidance for dental clinics were available from OSHA and professional organizations such as the American Dental Association, little is known about the real-world application of these recommendations.^2,7,8^

Limited data have been published documenting how the COVID-19 pandemic affected DHCP usage of PPE and how DHCP adapted their clinical practices to protect themselves from COVID-19.^5,9–11^ The purpose of this study was to characterize PPE use during the COVID-19 pandemic and to develop and pilot test an educational video on proper PPE donning and doffing procedures for use in dental clinics.

## Methods

### Study design and recruitment

This study was a qualitative analysis of 2 sets of semistructured interviews. The study included DHCP from 8 dental clinics in a Midwestern metropolitan area: 3 community-based general clinics, 3 community-based pediatric clinics, an academic clinic, and a hospital-affiliated pediatric clinic. Clinics were recruited through snowball sampling. DHCP with clinical care responsibilities and experience using PPE were eligible to participate. The study took place from September through November 2020 and from March through October 2021. Participants provided written, informed consent. The Washington University Human Resources Protections Office and the St. Louis University Institutional Review Board approved this study.

### Interview 1

*Donning and doffing PPE.* This study was performed in conjunction with an assessment of donning and doffing PPE methods.^12^ After donning and doffing, individual semistructured interviews were conducted in person to characterize PPE use during the COVID-19 pandemic. The interview guide and questions are summarized in Appendix A.

*Donning and doffing educational video.* Qualitative and quantitative data from the donning and doffing interview were reviewed and analyzed to develop an educational video tailored to DHCP.^13^ This video demonstrated donning and doffing in accordance with US Centers for Disease Control and Prevention (CDC) guidance.^14^

### Interview 2

*Video feedback.* After reviewing the educational video, DHCP participated in individual and group semistructured qualitative interviews to obtain feedback on the relevance and usefulness of such a tool for DHCP. Interview questions are summarized in Appendix B.

#### **Assessment**

Audio files were transcribed and coded independently by 2 study members trained in qualitative research methods. Thematic patterns were identified using deductive coding. Discrepancies between coders were resolved through discussion. Study team members selected representative quotations from common themes for the results.

## Results

Participation included 70 DHCP in the donning and doffing interview and 62 DHCP in the video feedback interview (48 DHCP participated in both). Demographic and occupational background were available for the 70 participants who participated in the donning and doffing interview. Ages ranged from 19 to 70 years, and 78% were female. More than half (54%) reported ≥5 years of clinical experience, and 23% had ≥10 years of clinical experience. Dental assistants and hygienists accounted for 54% of participants; dentists or dental residents accounted for 46%.

### Interview 1: PPE donning and doffing practices

Interviews after donning and doffing were coded into 6 thematic domains. Interviews are summarized in Table 1.

**Table 1.** Themes and Quotes From Donning and Doffing Interviews

Themes	Quotes
Domain 1 Prior personal protective equipment (PPE) training	“We learned a little bit [about donning and doffing] in dental school, but I don’t feel like it was one of those things that was all that reiterated, I think the biggest area that I learned was in the operating room.” “We did do this orientation at the beginning of residency right before we started seeing patients in the clinic. It was really for all the medical residents, including dental, we kind of went through…the same thing. We watched videos on how to don and doff PPE and then in dental school we used to do things, not an activity like this, but watched videos on how to properly wear PPE, but it’s been a while, so I did forget the order of things.” “Here [place of employment], …they didn’t actually teach us, so I had to kind of improvise here and I had to think back to what I had been doing in dental school and they did teach me […] I’m sure there was a whole course and online module, and someone showed us and people constantly reinforced it.” “We did 2 sessions in the classroom… In the clinic we did a session with the head of infection control in the dental school, and then we were audited periodically as well.”
Domain 2 Changes in PPE use due to COVID-19	“Well, before COVID, it was always just a surgical style and… At school, whenever COVID happened it was N95.” “I definitely like the goggles over the face shield because I feel like I can see easier [sic]. I have better access to seeing the patient; I think the face shield kind of makes that tough. I also get a lot more hot and sweaty when I have the face shield on…” “[I would double mask] If I’m feeling under the weather or I notice on a note that a patient has had COVID-like symptoms…” “We started using the face shields; we weren’t using those before. A lot of people use the side additions to glasses and safety glasses as before we just had regular glasses.” “I think the headgear is equally important… the head protector or, let’s say, the net protectors that we use – they really don’t do a good job. I personally bought some cloth-based covering and I feel better with those.” “We use them [N95s] 3 times before I dispose them, but the way I use them I use a regular mask on first, use my N95 mask, and then put a regular mask on top. So I used 2 of these masks, but the one N95 is in the middle.” “We were actually utilizing N95s and then they made us put like a surgical mask or a level 3 mask over that. So we were actually disposing [the surgical style masks] and keeping the other N95s over 5-time usage.” “It was pretty confusing in the beginning, nobody knew exactly what we needed to do as far as precautionary measures, so let’s grab the whatever you can and use what you can. And I guess the ADA recommendations that were provided to us was we want at least an N95… so we thought, you know we could keep the N95 intact as long as we possibly can by wearing another mask on top of that that would switch between patients. Face Shields because of aerosols. But at the end of the day, it was whatever we’re eventually comfortable with, and I was a little more on the precautionary side so I took all of this throughout the entire process from March to August… At the beginning [of COVID] it was following ADA recommendations and then at the end it was still following the recommendations, but doing whatever I felt was more comfortable for my own safety, which was keeping everything on because I don’t care how much of a hassle it was, I still did it nonetheless.”
Domain 3 Challenges to obtaining PPE	“Ordering gloves has been hard for us; we don’t have any mediums right now ‘cause [sic] they’re on back order… A lot of girls in the office wear mediums, and so we’re all kind of having to squeeze into smalls to make it happen.” “I use this scrub jacket. We were not able to get gowns early on and we just kind of adapted to this.” “We had short supply of the N95[s] so I used these [N95] for about a week to 2 and then I put an extra surgical mask over the top and I switch that out between patients.” “Well in the beginning it was just hard, for our office as a whole. It was hard getting their hands on the KN95 masks and stuff like that for sure. Gloves were on back order for a while, Cavicide wipes…” “We had problems getting N95s. Once we got KN95s they were not approved so then we had to ship those back then they said they wouldn’t take them back, then we spent thousands and thousands and thousands of dollars. Gowns. The gown cost [sic], the gowns took forever to get, then they were delayed… We did get gowns 7 months later.” “It’s always been steps of hardness of things to get. Cavicide has been sold out. We were looking at some hydrogen peroxide, different materials that we would not normally use because it’s been completely impossible to find anything.” “We were running extremely short [on N95s] to the point where we couldn’t treat people sometimes.” “The N95s. There’s a time we were reusing them. We went up to 5 times using. They were cleaned every day at the end of each day… Over time it just got a little annoying because …the strap would start to break or things like that, but mostly wasn’t too bad.” “We couldn’t get ahold of gowns, I think, at the beginning of COVID so we didn’t see any point in wearing it.” “The CaviWipes, as far as I know, I think we make our own sterilization wipes. I don’t know what chemicals they use but we no longer use the same CaviWipes we used to use anymore … I think the assistants make the wipes on their own using chemicals so, I mean, if we can go back to using the CaviWipes it would be nice because I used to wipe my safety goggles with CaviWipes before COVID but now I just do it with alcohol wipes just because we don’t have the CaviWipes we typically use anymore and we just have these wipes our assistants put together.”
Domain 4 PPE discomfort and compatibility with specialty equipment	“I understand these PPEs are here to protect us, but we put so much on that you almost hamper yourself from moving properly or seeing properly. I think there’s…a fine line between how much should I have and how much shouldn’t I have… I didn’t really like wearing the face shields so I didn’t really wear them very often.” “The [face] shield fogs and it also changes the focal length with the different material ‘cause [sic] you’re going through 2 different materials, and so you know to find the right spot is significantly harder.” “[I wear a] KN95, just because of the research says that right now, during COVID, that helps the best, even though I’ve had both of my vaccines. I’d rather have an N95, but the problem with the N95 is that they restrict my air way too much. I’ve actually used an oximeter with different masks, so I chose the one that I actually have 98 or above on.” “I can’t use my lights with the face shield on. Fortunately, our hand pieces do have lights on them, otherwise I would probably have to ditch the face shield, like I know a lot of dentists like one of our partners… he doesn’t use the face shield because he needs to use loupes.” “[When wearing loupes] The shield fogs and it also changes the focal length with the different material ‘cause [sic] you’re going through 2 different materials, and to find the right spot is significantly harder.” “The KN95’s are thicker, they sit up higher, and [it] changes the focal length [of my loupes] which was made specifically for me without wearing a KN95 years ago. So I have to change the height of the patient which can sometimes compromise just my stature and bending over or whatever.” “[On not using eye protection] We worked through a microscope and it wasn’t possible to see through the scope because it is too far back so here at [location] we didn’t do any modifications, but other endodontists have just hooked the face shield onto the scope so that it’s fixed on the scope. We didn’t do that here.” “We use the microscope. If you wear glasses, it’s hard to get close enough to be able to really see through unless you have like smaller glasses. The goggles are too thick to really get up close enough to see everything you see so typically, I haven’t used them.” “I typically used to [wear loupes], but since COVID I have stopped using loupes because there’s just nowhere to fit them with the face shield and mask…if that would get fogged up there was no way that I could defog it during the procedure.” “So when we first started dental school, they brought us in to fit us for these masks and basically what happens is you know you have that metal piece on the N95 mask that sits off the bridge of your nose maybe 2 to 3 millimeters. And if you’re wearing loupes, well now the distance your field of view and the distance that you have is now changed compared to what they measured so that definitely changes it. It actually changes your magnification in your loupes, so you get a little bit of distortion naturally,” “The difficulty was the face mask fogged up quite often, and if you didn’t get a good seal with your N95 or Level 3 sneaked up into the loupes, that either can block your vision or could clog up the loupes and now you have double problems.” “[Face shields that work with loupes]… there’s a light on it [the loupes], and it [face shield] will hit the light a lot of times, so sometimes they make specialty ones that have a little cut out that the light can stick through on the face shield.”
Domain 5 PPE reuse	“Because we have limited amount of N95s available in the clinic I would wear my N95 and wear this [surgical style mask] over. Then I would do crowns and fillings; then I would throw out the outer [mask] and put on a new mask. That way I’m reusing the N95 for different patients.” “We had N95s that we, because of the shortage at the time, had to end up reusing them. We used them once a day for the week… and then we carried them over to the next week and… I believe we use them that way for a 3-week period then we disposed them.”
Domain 6 Additional precautions outside PPE use to protect DHCP	“I think we make our own sterilization wipes. I don’t know what chemicals they use but we no longer use the same CaviWipes we used to use anymore. I think the assistants make the wipes on their own using chemicals.” “Just temperature and questionnaires, but we actually did have we had an employee… She came in on Monday, saw patients Tuesday. She came in and she had a temperature. So we sent her home… Turns out she had COVID. We contacted the patients that were seen by her so there about 7 patients for the whole day that were seen by her on Monday, told them to monitor their symptoms and not a single one had any issues, so I feel like it’s working pretty well, hopefully.” “We’ve just practiced social distancing … especially from the beginning, they had us not eat in the break rooms and just… going out to our cars to eat and washing our hands a lot, making sure that personal belongings… normally we wouldn’t have them on the table and then keep them in your locker. I’m just trying to practice like that kind of stuff. Not coming to work if you don’t feel well…” “We tried the social distancing—that’s really tough for us… I was a little upset whenever they said we didn’t have any studies for 6 feet, 3 feet may be fine… I would say that when we first started, we did have a conscious, conscientious effort to try to stay 6 feet apart to during lunch. You know, make for sure that not everybody was in the lunchroom. Other people were eating in their cars. That effort has since waned because a lot of these guys have gotten their vaccines as well.” “I was doing the floors—cleaning the floors after each op, changing the time between ops [ie, adding more time between ops]… separating [physical distancing] that includes… the waiting room. How often patients are coming in and out. I can’t think of anything else besides washing the floor” “The air cleaning units are interesting. It’s just one of those things where I don’t know whether or not it’s going to be like gloves before AIDS where people are like I can’t believe we didn’t do this before or if it’s going to be like a big money scam from people like where yeah, in theory it works. I mean it’s going to decrease the viral loads, bacterial loads, and it makes sense, but I just don’t know how you go from zero to all of a sudden. It’s all over the markets and, what’s good and what’s not good?” “Now I wear a hairnet to cover my hair to prevent me carrying splatter or anything in my hair… I wash my hands more frequently than I used to. In dentistry, we frequently all wash our hands; I do it more often now than usual. I try my best to always have on gloves at all times when interacting with patients even if it’s just taking vitals I put on gloves now… and [how] I clean up instruments has changed… Certain things we only had to wipe down with Cavicide wipes, we’re now shaking in an ultrasonic cleaner.” “We were using mouth rinses for a time, routinely. One time we were just using chlorhexidine and then I think they did have some hydrogen peroxide that we were using as well but mostly just chlorhexidine mouth rinse pre-op.” “They use air purifiers in the rooms. I think that was something they added. They added barriers in the front of the offices. Plexiglass, and then before that it was also the people in the front desk also wore face shields for new patients that were coming in, we did all the cautionary measures… we screened all the patients before they came in. Phone calls. Went through the checklist and all that.”

*Domain 1: Prior PPE training.* Overall, 60 (86%) of 70 DHCP reported receiving some prior PPE training. Among them, 36 (51%) received PPE training on the job, and 31 (44%) had received training in residency or other formal education. Only 10 (14%) of these 70 DHCP had received this training through an educational video. One DHCP said, “We did this orientation at the beginning of residency right before we started seeing patients in the clinic. It was for all the medical residents, including dental, and we went through kind of the same thing; we watched videos on how to don and doff PPE.” Similar experiences with previous PPE training were reported among 27 (84%) of 32 dentists and dental residents as well as 28 (85%) of 33 dental assistants and hygienists. Of the 61 DHCP who had received prior PPE training, 13 (21%) had received multiple forms of training.

*Domain 2: Changes in PPE use due to COVID-19.* DHCP reported increased use of N95 respirators and changes in eyewear due to COVID-19. When asked about mask use, 59 (87%) of 68 DHCP reported using N95 respirators. For example: “Before COVID, it was always just a surgical style [mask] and… whenever COVID happened, it was N95.” Among DHCP asked about N95 respirator fit testing, 24 (71%) of 34 reported having undergone formal fit testing. Fit testing was significantly less common among community DHCP compared to academic and hospital DHCP (47% vs 100%; Fisher exact *P* < .01).

Among these 70 DCHP, 37 (53%) reported wearing eyewear that included face shields, 36 (51%) wore safety glasses or glasses, and 14 (20%) wore goggles; 22 (31%) reported using multiple forms of eyewear. Of the DHCP who wore eyewear, 45 (71%) of 63 wore eye protection that met CDC guidelines. DHCP reported choosing eyewear based on factors such as size and compatibility with specialty equipment. One DHCP said, “I definitely like the goggles over the face shield because I feel like I can see easier [sic]. I have better access to seeing the patient; I think the face shield kind of makes that tough.”

*Domain 3: Challenges to obtaining PPE due to COVID-19.* Early in the COVID-19 pandemic, DHCP experienced challenges with obtaining PPE, including gloves, disposable gowns, and N95 respirators. One DHCP said, “Ordering gloves has been hard for us; we don’t have any mediums right now ‘cause [sic] they’re on back order… A lot of girls in the office wear mediums, and so we’re all kind of having to squeeze into smalls.” These challenges also affected what PPE DHCP used. One DHCP said, “I use this scrub jacket. We were not able to get gowns early on and we just kind of adapted to this.”

*Domain 4: PPE discomfort and compatibility with specialty equipment.* DHCP experienced difficulties using PPE, including compatibility with specialty dental equipment and physical discomfort. When prompted about challenges wearing PPE, face shields were commonly mentioned due to size, incompatibility with other equipment, and interference with mobility or ability to see. One DHCP said, “I understand these PPEs are here to protect us, but we put so much on that you almost hamper yourself from moving properly or seeing properly… I didn’t really like wearing the face shields, so I didn’t really wear them very often.”

Among DHCP asked about specialty equipment, 28 (46%) of 61 reported using items such as loupes, light attachments, or microscopes. Among the 28 DHCP, dentists (24 of 28, 86%) were more likely to report using specialty equipment than dental assistants (4 of 28, 14%). Of DHCP who used specialty equipment, 17 (61%) of 28 said PPE interfered with their ability to see and use equipment appropriately. On discussing loupes and face shields, one DHCP said, “The [face] shield fogs and it also changes the focal length with the different material ‘cause [sic] you’re going through 2 different materials, so… to find the right spot is significantly harder.”

DHCP reported challenges with wearing an N95 respirator. Physical discomfort was a commonly reported factor for not wearing an N95 respirator. One DHCP said, “[I wear a] KN95… I’d rather have an N95, but the problem with the N95 is that they restrict my air way too much. I’ve actually used an oximeter with different masks, so I chose the one that I actually have 98 or above on.” Another DHCP mentioned that improper N95 respirator fit led to fogging of loupes during use.

Some DHCP eliminated use of specialty equipment all together. One DHCP said, “I typically used to [wear loupes], but since COVID I have stopped using loupes because there’s just nowhere to fit them with the face shield and mask… if that would get fogged up there was no way that I could defog it during the procedure.”

*Domain 5: PPE reuse.* A variety of methods were used to extend PPE use. For example, 26 (44%) of 59 DHCP who used N95 respirators also mentioned sterilization techniques, such as ultraviolet (UV) light, autoclave, and use of a baby bottle sanitizer machine. Many DHCP also reported wearing a surgical mask over their N95 respirator to preserve and facilitate its extended use or reuse. One DHCP reported, “I would wear my N95 and wear this [surgical mask] over. Then I would do crowns and fillings; then I would throw out the outer [mask] and put on a new mask. That way I’m reusing the N95 for different patients.”

*Domain 6: Additional precautions.* Temperature checks and health screening questionnaires were most often used for both patients and DHCP (14 of 67, 21%). Additional precautionary measures implemented included rinsing a patient’s mouth with antiseptic, more frequent sanitization of floors and surfaces, installation of air purifiers and enhanced ventilation, and making homemade disinfectant wipes in the clinic. One DHCP said, “We were using mouth rinses for a time, routinely. One time we were just using chlorhexidine and then I think they did have some hydrogen peroxide that we were using as well, but mostly just chlorhexidine mouth rinse pre-op.”

### Interview #2: Training Video Feedback

Overall, 62 DHCP provided feedback on the PPE training video, most of whom responded positively. One DHCP shared, “I thought it [video] was informative with the equipment that we were given. It’s pretty consistent with what we experienced in dentistry in terms of recommendations; I think it was spot on.” Responses were coded into 4 thematic domains. Training video feedback is summarized in Table 2.

**Table 2.** Themes and Quotes From Video Feedback Interview

Themes	Quotes
Domain 1 Exposure to similar video	“It was a component of…a blood borne pathogen video, so they would talk about how to protect yourself, and one of the components was both the donning and doffing. I think the main difference that I noticed is in those videos they talk about the whole process and then there’s a video at the end with a separate link. I like how they incorporated here, here’s step one and then they show you how to do it. Here’s step 2 and they show you how to do it. So I thought that was pretty helpful and keeps it fresh in your mind.” “No, that was actually my first video, that I could actually see how like the proper ways to do all that.” “My instructor, we watched the video and then she showed us all [how to don and doff] and then we all did it.” “No, I went to… [a] program down in [city], but they kind of went over on how to like take off PPE and apply it and do it properly. I feel like this was a lot more beneficial and efficient.” “I don’t think that we have ever had as far as… training, specifically on donning and doffing equipment.” “I don’t think that I’ve seen any videos like that in the past. I know that there have been conversations… in my previous places that I’ve worked about donning in doffing, but I’ve never seen the video showing and walking me through how to do it.” “I think there was a live a demonstration [of PPE] with the nurses before going to the OR… I can remember seeing someone do it, I know it was not a video or it was pictures… like a series of pictures.” “Yeah, in dental school, the head of infection control—before we got into the clinic and also… our first year—she did a classroom-based thing with us where she showed a video. She also did it in person and we did it hands-on in the classroom… and then we were audited periodically throughout our time in dental school.” “I remember it was both… like a PowerPoint and then also like someone did the explanation while someone [else] donned/doffed equipment… Oh yeah, it’s [watching a video] a lot easier. It’s way easier than…watching a PowerPoint; you actually get to see someone do it.” “At the beginning of our residency orientation, right before we started seeing patients in the clinic, we had this orientation that was for all the medical residents, including dental and we I remember talking about… how to put on the PPE… take it off. But I don’t really know if we watched a video like this… this video was definitely a good refresher for me.” “I’m pretty sure we had a video in the military that then we did clinical training afterwards. We did so much training, I don’t remember 100%.” “[Place of employment named] gave a whole rundown on how to put on and take off PPE in the hospital that was good… but not in a dental setting.” “Pretty similar video with my first job. They presented something vastly similar. It was either with the ADA or the CDC, I don’t remember.” “We had some training, but also when I was at my job when COVID hit, they did some extra training for instructional videos. Kind of followed suit with everything that was going on.”
Domain 2 Knowledge gained	“It [performing hand hygiene] makes sense in between when you take the gown off that you use hand sanitizer. I usually do that, but then I see that they, between taking the mask off, they washed or hand sanitizer again. I thought that was interesting.” “I typically don’t roll my gown [when doffing], and that’s actually where I picked up some fluorescence on my hand when I was taking off my gown. I must have touched the outside surface somewhere.” “You covered it in your video, but I think it is tricky for dentists ‘cause [sic] we have loupes… you want to make sure you’re not using contaminated gloves or sometimes it [loupes] slides down while you’re working. You’re going to like, [touches bridge of nose] push it back up.” “I feel it’s very accurate that they said the face shield is what you should use and that glasses are not an acceptable alternative.” “I didn’t know really that that was called donning and doffing… I had no idea that was a thing at all.” “I think this was a little bit more up to date as far as… PPE … [compared to “If Saliva was Red”] because we didn’t have the face shield. So we had gowns, but… they worked a little bit differently back in the early 80s… If I remember the video correctly, they didn’t go over the exact sequence on when that should happen as far as taking them off.” “I don’t think it showed me anything I was unfamiliar with, but I think it’s always like a good reminder on how to do it the right way.” “That… [video] was super helpful as far as… you taking your gloves off first and then hand sanitizer and then not touching goggles without sanitizing…” “I definitely learned stuff… well, I learned I was supposed to use the sanitizer in there [ie, during doffing]. I didn’t realize that was… meant to be part of everything.” “Well, I learned that I don’t always do it correctly. If we’re being honest about it.” “I don’t think I was removing my PPE as I should. So, I think I learned how to do that…” “I thought it [video] was really informative. One thing that stood out to me was taking off my glove before I remove like the body [sic] shield ‘cause [sic] usually I just kind of rip it off and then I take off my gloves, so that’s one thing that kind of stood out to me, and then also using the hand sanitizer after I’m completely de-gloved and then removing my… eye and headwear.” “Yeah, I would say mostly just the hand sanitizer in between… Usually I just save it for at the very end after I’ve degloved [sic] and everything… but I see that they used it twice during the de-gowning process. So that’s something that is kind of new for me.”
Domain 3 Relevance to clinical practice	“I thought it [training video] was informative with the equipment that we were given. It’s pretty consistent with what we experienced in dentistry in terms of recommendations; I think it was spot on.” “It’s just helpful to get everyone on the same page… if your whole office or all of your residents are together could watch and if you’re all doing the same thing over and over again, then you’re more likely to make that a habit, so I think having a video that walks us all through doing the same thing would be helpful.” “The main difference is that we’re using reusable or washable gowns, like a lab coat. As far as everything else, taking on and off, it’s [the educational video] pretty consistent…” “I think it [the educational video] covers a very broad, very good group of practitioners. Periodontists don’t do hospital rotations anymore, we’re not scrubbing into the OR like we used to. So oral surgery is kind of on their own as far as that goes. They have more strict procedures than any general dentist will ever do.” “I like how they incorporated here, here’s step one and then they show you how to do it. Here’s step 2 and they show you how to do it. So I thought that was pretty helpful and keeps it fresh in your mind.” “[Regarding loupes] I don’t know what study would need to be done or what would be looked at for that, but you had that little snippet at the end which I thought was really unique.” “[Regarding onboarding] We usually just use what’s on [institution training website], but we do actually have a new person starting in the next couple weeks so, that video would definitely help.” “And I like that it’s not too long. I think it’s short and to the point. I think that it’s very helpful. It keeps their attention.” “Yeah, I think it’s definitely important because… even if it’s just on the job training, they [employees] should always have something like that, you know, and I don’t think that we even have something where… we’re going to take the time to specifically focus on how to take off your PPE and put it on, so I think it definitely needs to be implemented.” “I think for someone, especially who’s new to the field, I think it’s very helpful because it’s kind of [a] big task trying to figure out how to get all that on the proper orientation.” “I actually think the discussion about… about not touching them [loupes] with your gloves and then also not having them rest on a dirty gown – that’s very valid and something that we… struggled with in dental school.” “I definitely think… a refresher course would be helpful to build something that you can just kind of go to, or maybe at the start of each like clinic year or residency year and just kind of have that available.” “I think that this training is always good to be refreshed… because I feel like dentists oftentimes… they get there in a hurry, so they just take off their gloves, leave the mask on when you take it off, and then on to the next patient… and so I feel like reminding them and kind of seeing that what you could be contaminating and what you could be passing along is important. “But it’s [learning how to don and doff] a lot like CPR. You learn it, you know it, but you forget it. You skip steps, take shortcuts, you just don’t even think about it. I think it’s valuable to have that information reviewed with you periodically, even if it’s an abbreviated format. Or spelled out on a list that’s posted.” “Right, I think for clinical if you’re at a school or academia, I think it’s more fit to watch something like this because the patient load is much smaller. There’s less—there’s more time that you have to work on your patients or work between patients. Rather, I think a different video would be needed for private practice. Specifically for the things that I brought up earlier, like you’re moving between patients, you doing hygiene checks and stuff like that is this feasible between all those patients?” “I think a lot of dentists… I think the vast majority follow pretty strict protocol as far as PPE, but it’s the assistants and support staff that could use more [training]. Any office I’ve gone into you’ll see the assistants and think ‘what are you doing?’.”
Domain 4 Additional information that would be helpful	“The only thing I would like to see in the video is the N95 for if you were to double mask for a certain reason, what the protocol would be for that…? Personally I will put on an N95 and then I’ll put a mask like this on over. Should I—you know mostly for the removal process—How would that work? Should I take off the top one and then hand sanitize and then take off the lower one? Or should I take them both off at the same time or something like that?” “I think there’s 2 points that I wanted to highlight… (1) They said you’re not supposed to hold your loupe while you’re working and that’s kind of not realistic … because we need to like adjust the light. So we just use them and then clean it with the CaviWipe, usually after. It is kind of impossible to just not to use them, it’s just not realistic. And the other thing… I think a hair net is kind of essential. ‘Cause [sic] once you’re doing [osseous] surgery, there’s going to be blood everywhere and… patients as you said, sneeze, cough, all of that stuff—bloody stuff—that may come to your face.” “I think a hairnet should be, I think, at least for my specialty it should be included. And nobody pays attention to that… As I said, it’s not mandatory to use it, but people like me and my co-residents usually use it in surgeries… It’s a disposable one and we just take them off as soon as we’re done. And… I’ve seen blood on it before.” “I guess sometimes we wipe our own equipment. For instance, you know we wear our loupes… Do you want us to change into a new gloves when we wipe our contaminated eyewear.” “The video looks like they didn’t put on the loupes and they said CDC or whoever they don’t have a specific recommendation on that, so I guess I can just kind of squeeze it in as I see fit?” “I guess one critique… is implementing specialized dental equipment… I know there’s no CDC recommendation for loupes and stuff like that. I think that would be very helpful if they include that in there. Just because I would say, like, 90% just general dentists would be using them just patient to patient. With that in mind as well…but if you wanted to apply to private practice, I think that idea would be also implementing something that would recommend doing hygiene checks. But otherwise I think in terms of just general heavy equipment for the dental field I think it was good.”

*Domain 1: Exposure to similar video.* When asked about previous exposure to an educational video, 26 (43%) of 60 DHCP reported having seen a similar video. One DHCP said, “I think the main difference that I noticed is in those [other] videos they talk about the whole [donning/doffing] process and then there’s a video at the end with a separate link. I like how they incorporated here’s step one and then they show you how to do it.”

Of the 26 DHCP that reported exposure to a similar educational video, 11 (42%) cited “If Saliva Were Red,” a dental infection control and safety educational video created by the Organization for Safety, Asepsis and Prevention.^15^ One DHCP said, “I think this was a little bit more up to date as far as… PPE… [compared to “If Saliva Were Red”] because we didn’t have the face shield… If I remember the video correctly, they didn’t go over the exact sequence on when that should happen as far as taking them [PPE] off.”

*Domain 2: Knowledge gained.* Common feedback included increased knowledge regarding hand hygiene frequency and the proper sequence for doffing PPE. DHCP were familiar with practicing hand hygiene prior to doffing PPE but were less familiar with performing hand hygiene between individual doffing steps. One DHCP said, “It [hand hygiene] makes sense in between when you take the gown off that you use hand sanitizer. I usually do that, but then I see that they, between taking the mask off, they washed or hand sanitizer again. I thought that was interesting.”

After reviewing the video, some participants identified where they might have deviated from protocol in the initial donning and doffing assessment.^12^ One DHCP stated, “I typically don’t roll my gown [when doffing], and that’s actually where I picked up some fluorescence on my hand when I was taking off my gown.”

*Domain 3: Relevance to Clinical Practice.* When prompted about relevance to clinical practice, certain PPE items were identified as not applicable, most notably disposable gowns. One DHCP said, “The main difference is that we’re using reusable or washable gowns, like a lab coat. As far as everything else, taking on and off, it’s [video] pretty consistent.”

Among the 58 DHCP who had a positive reaction to the video, 18 (31%) also mentioned that the video would be useful during onboarding for new employees and/or as a part of annual training. One DHCP said, “It’s just helpful to get everyone on the same page… I think having a video that walks us all through doing the same thing would be helpful.”

*Domain 4: Additional information that would be helpful.* When prompted about video improvements, 16 (30%) of 53 DHCP expressed need for additional guidance. One DHCP stated, “The only thing I would like to see in the video is the N95 for if you were to double mask for a certain reason, what the protocol would be for that… Should I take off the top one and then hand sanitize and then take off the lower one? Or should I take them both off at the same time or something like that?”

DHCP suggested more information is needed on how to don/doff specialty equipment safely. One DHCP said, “I guess one critique… is implementing specialized dental equipment… I know there’s no CDC recommendation for loupes and stuff like that. I think that would be very helpful…”

## Discussion

This real-world study captured dental challenges and concerns throughout the COVID-19 pandemic, including before and after vaccines became available. DHCP expressed significant challenges with PPE regarding supply and usage; this is consistent with the findings of Tabah et al,^16^ who noted that DHCP experienced PPE shortages and needed to reuse single-use PPE items. As a result, DHCP modified PPE to accommodate their needs. DHCP and clinics included in this study adapted to COVID-19 with resourceful solutions; however, the impact of these changes on PPE effectiveness and durability are unknown.

DHCP commonly faced challenges related to respirator use and reuse. Methods for extended N95 use (ie, wearing the same respirator for multiple patient encounters) may reduce effectiveness of PPE.^2,17^ CDC guidance states that extended N95 respirator use is not ideal for conventional everyday practice, but it can be done in cases of expected and known shortages.^17^ In response to limited supply of N95s, DHCP adopted methods for extended use, including cleaning and double masking or wearing a surgical mask over an N95 respirator.

In addition, fit testing was not universal among the DHCP in this study. Fit testing is required by OSHA; however, enforcement discretion was applied temporarily during COVID-19.^18,19^ In our study, 24 (73%) of 33 DHCP who reported using N95 respirators received fit testing, whereas Tabah et al^16^ found that half of healthcare personnel had never undergone fit testing. Data regarding fit testing were not captured for 26 (44%) of 59 DHCP who wore N95s, suggesting that fit-testing rates may be similar to those of healthcare personnel. All 9 DHCP who were not fit tested were associated with community-setting clinics. Limited access to fit testing is a barrier to proper PPE use among community-based DHCPs. KN95s were not commonly mentioned, but DHCP sometimes used the term KN95 interchangeably with N95. This finding may be linked to limited prior N95 respirator fit testing experience because these tests are often accompanied by education of users on respirator specifications.

PPE effectiveness is complicated further by use of specialty equipment. Additional guidance is needed on how to properly don and doff specialty dental equipment and how to disinfect them between uses. Protective eyewear presented a physical barrier to the use of loupes and microscopes. Similar challenges have been reported in the field of ophthalmology.^20^ These challenges may limit use of specialty equipment or increase self-contamination. Recommendations on alternative protective eyewear compatible with loupes and microscopes are needed. Development of protective eyewear that physically incorporates loupes into the design may also serve useful.

DHCP found the educational video helpful and relevant to clinical practice. Educational videos are an effective training tool for teaching and learning, most notably as a supplement to in-person facilitation or demonstration for clinical based skills.^21–23^ Data suggest that incorporating an instructional video along with independent or observed practice of an instructor may be an effective method to teaching.^22,24^ Limited data are available regarding retention after PPE training videos; thus, recurrent training may be necessary as a component of continuing education. Future studies are needed regarding the impact of educational videos on postviewing PPE donning and doffing skills.

This study had several limitations. The sample size was relatively small and limited to 1 geographic region. Findings may not be representative of all DHCP due to sampling methods. Due to the nature of semistructured interviews, not all participants discussed the exact same topics. Furthermore, only 1 method of PPE donning and doffing was demonstrated in the video. Based on participant feedback, the incorporation of multiple CDC-approved methods would be beneficial in the future. Despite these limitations, this study provides valuable preliminary data on DHCP needs related to PPE and donning and doffing.

Similar to other healthcare personnel, DHCP faced challenges to applying PPE guidance due to supply shortages, specialty equipment, and variable levels of PPE training. Limited guidance may lead to unsafe or inappropriate PPE use; additional research is needed to improve occupational health and address unique challenges in dental care. Educational videos are an effective learning tool widely used throughout the healthcare field for clinical based skills and can help increase PPE knowledge among DHCP.

## Supplementary material

For supplementary material accompanying this paper visit https://doi.org/10.1017/ice.2023.6. click here to view supplementary material
