# Supplementary material for: Personal protective equipment use among dental healthcare personnel during the coronavirus disease 2019 (COVID-19) pandemic and the impact of an educational video in clinical practice
Source: Infect Control Hosp Epidemiol. 2023 Mar 16;44(9):1472–80. doi: 10.1017/ice.2023.6 (PMC10507497; doi:10.1017/ice.2023.6)
Supplement: Supplementary file 1 [file S0899823X23000065sup001.docx]

**Supplementary materials:**

**Appendix A**

*Interview 1: Personal protective equipment (PPE) donning/doffing practices qualitative questions*

1. Little is known about appropriate PPE in dentistry. Please describe what you typically wear and why.
   1. Probes: What kind of eye protection, masks, or respirators do you wear? Can you describe your preferences? What made you make that decision? If you use an N95 respirator, have you been fit tested?
2. How did you learn to put on and take off PPE?
   1. Probes: Who taught you how to use PPE? A supervisor? School?
3. Do you follow the same steps to take on and off PPE each time?
4. Dental workers often wear equipment unique to dentistry. Do you wear your equipment with your PPE? How do you do this?
   1. Probes: How often do you clean your equipment? What do you use to clean? Does PPE make this equipment harder to put on or remove?
5. Have you had any difficulties getting PPE? Have you had to reuse your PPE? Which items?
   1. Probes: If you reuse PPE, do you clean it prior to the next use? How do you store it?
6. Are there any additional precautions that you or your colleagues use to protect yourselves from COVID-19?
   1. Probes: Please tell me more about that. Is that practice common?
7. A final question: Are there any other issues with PPE in dentistry that we haven’t covered?

**Appendix B**

*Interview 2: Post donning/doffing video qualitative questions*

1. [After watching the video:] Have you seen any sort of training video like that previously?
2. Do you feel like the video was helpful?
3. Were the donning/doffing procedures in the video consistent with what you normally do in your clinical practice?
4. Is there anything that you would find more helpful or different?
